# Supplementary material for: Leptin Downregulates Angulin-1 in Active Crohn’s Disease via STAT3
Source: Int J Mol Sci. 2020 Oct 22;21(21):7824. doi: 10.3390/ijms21217824 (PMC7672602; doi:10.3390/ijms21217824)
Supplement: Supplementary file 1 [file ijms-21-07824-s001.pdf]

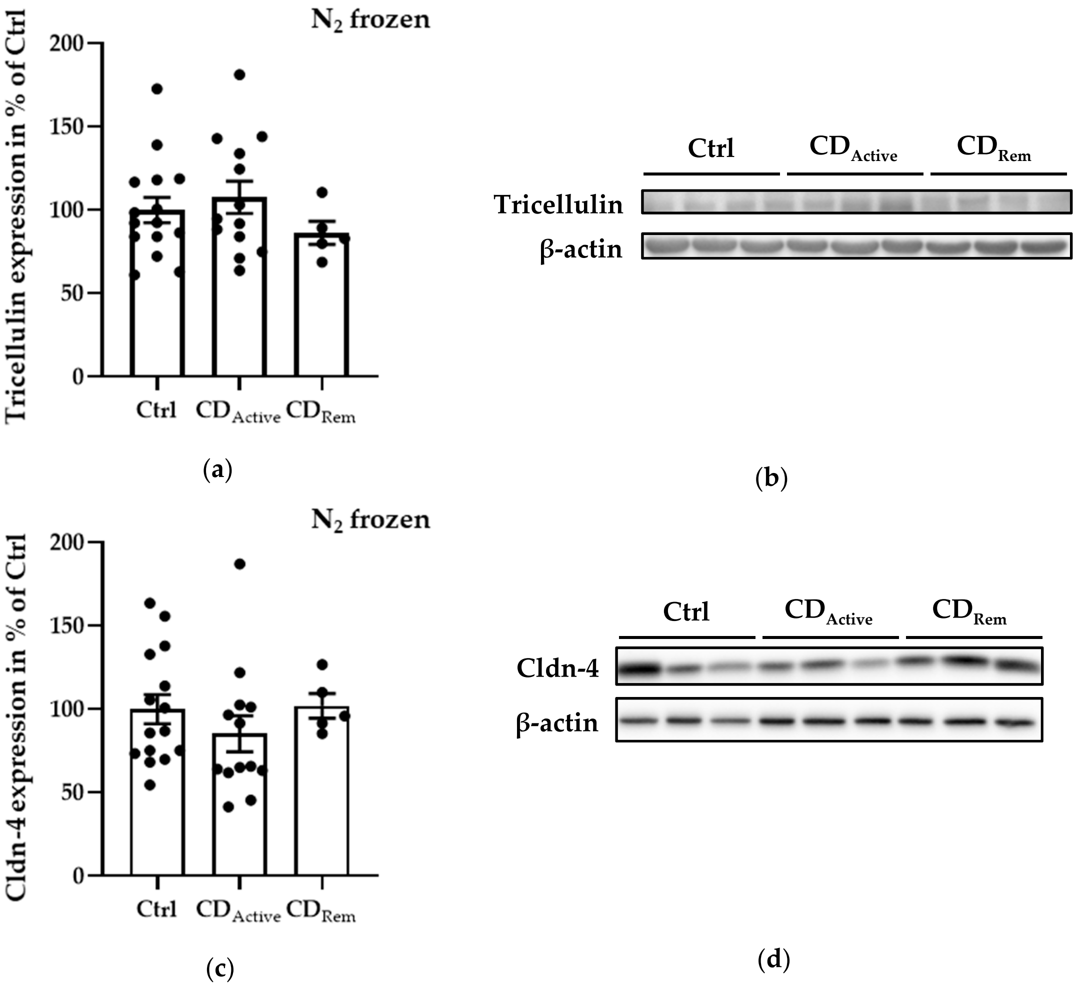

Figure S1. Protein expression analysis of tricellulin and Cldn-4 in human intestinal tissues. (**a**) Scatterplot with bar of tricellulin in Ctrl, active CD, and remission CD. Mean value of Ctrl is set to 100%. Ctrl: 100 ± 7.64 %, n = 15; CD_{Active}: 107.60 ± 9.70 %, n = 13; CD_{Rem}: 86.31 ± 6.93 %, n = 5. (**b**) Representative western blots of tricellulin. (**c**) Scatterplot with bar of Cldn-4 in biopsies of Ctrl, active CD, and remission CD. Mean value of Ctrl is set to 100%. Ctrl: 100 ± 9.24 %, n = 15; CD_{Active}: 85.23 ± 10.80 %, n = 13; CD_{Rem}: 101.98 ± 7.41 %, n = 5.

T84

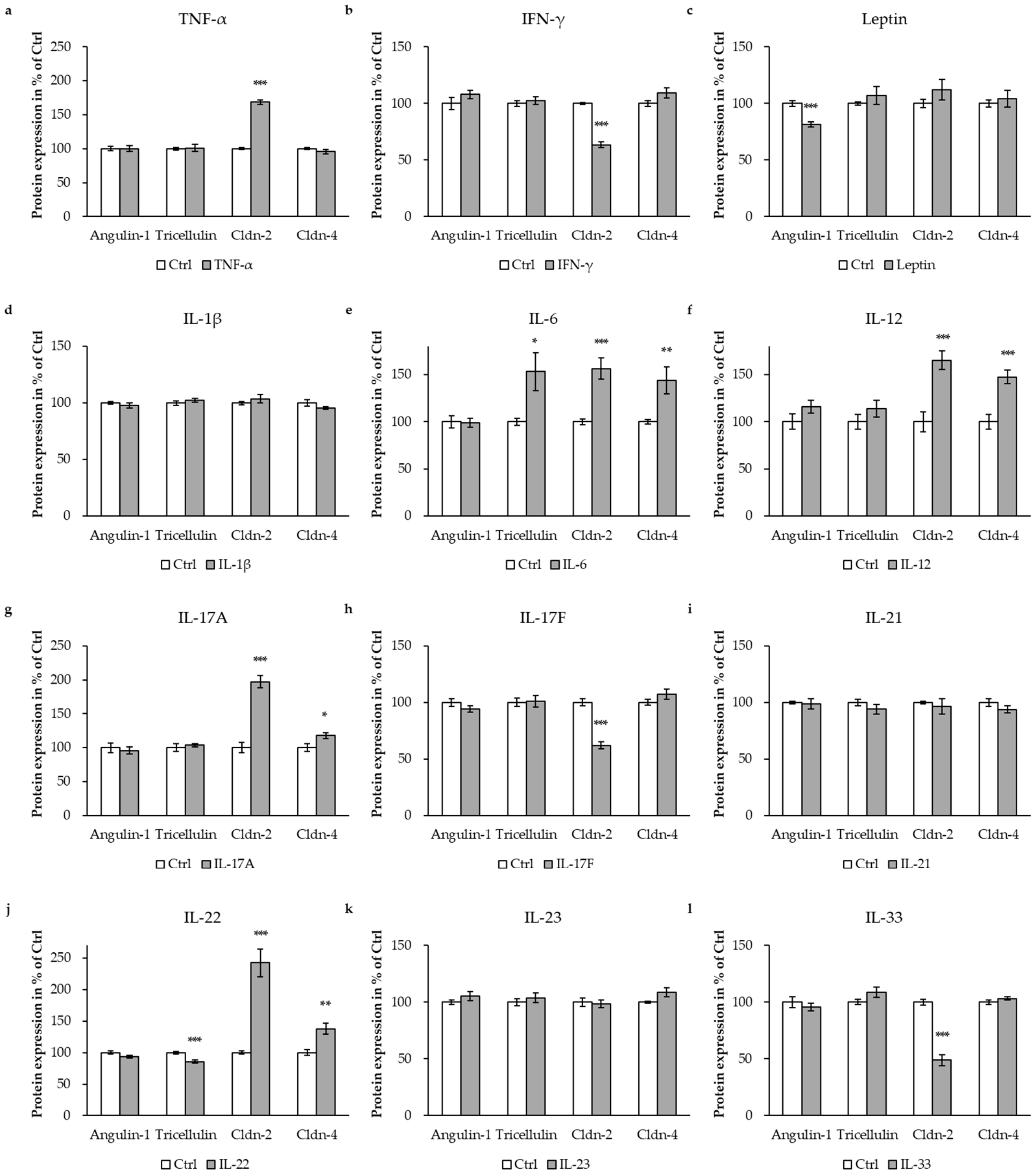

Figure S2 Cytokines effect on TJ protein expression in T84 cells. Densitometric analysis reveals that leptin leads to a downregulation of angulin-1 to 81.11 ± 2.07 % of untreated Ctrl after 48 h (c, ***$p < 0.001$, n = 12), while other cytokines do not affect angulin-1 expression. Tricellulin expression is reduced by IL-22 treatment for 48 h (j, ***$p < 0.001$, n = 12) while elevated by IL-6 treatment for 48 h (e, *$p < 0.05$, n = 12).

T84

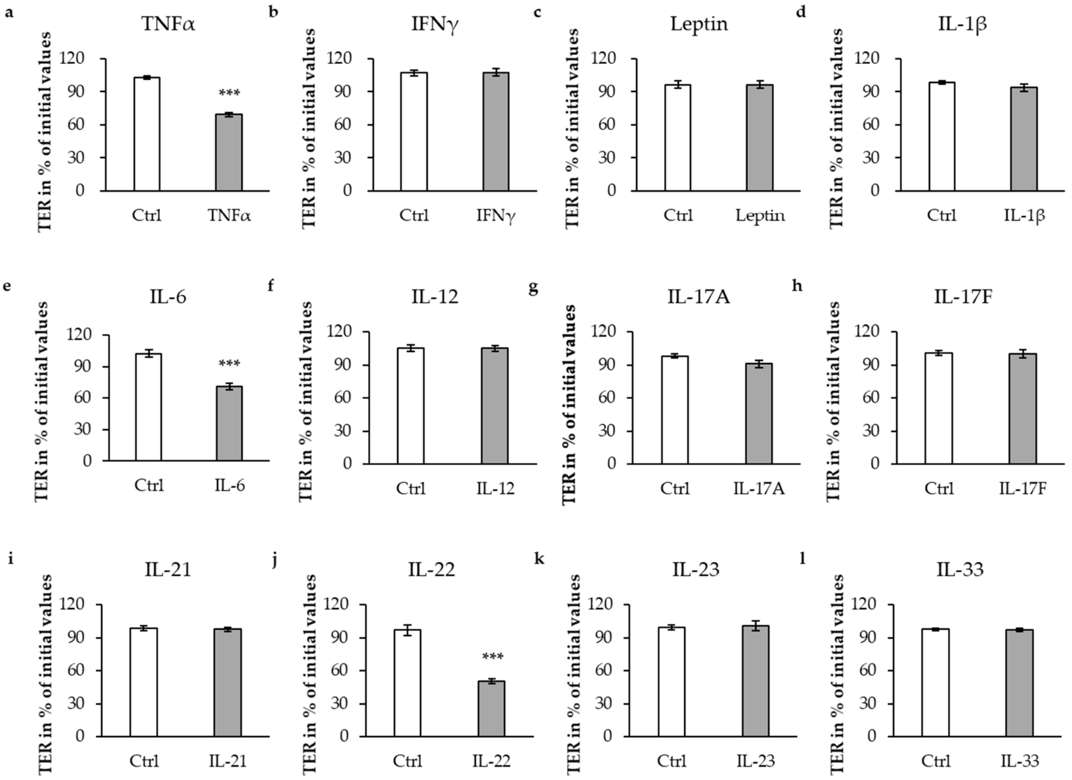

Figure S3 Cytokines effect on TER in T84 cells. 24 h of TNF-α treatment, 48 h of IL-6 or IL-22 reduces the TER to 69.37 ± 1.93% (**a**, ***$p < 0.001$, n = 9), 71.12 ± 3.45% (**e**, ***$p < 0.001$, n = 9), and 50.42 ± 2.22% (**j**, ***$p <$ 0.001, n = 9) respectively.

Caco-2

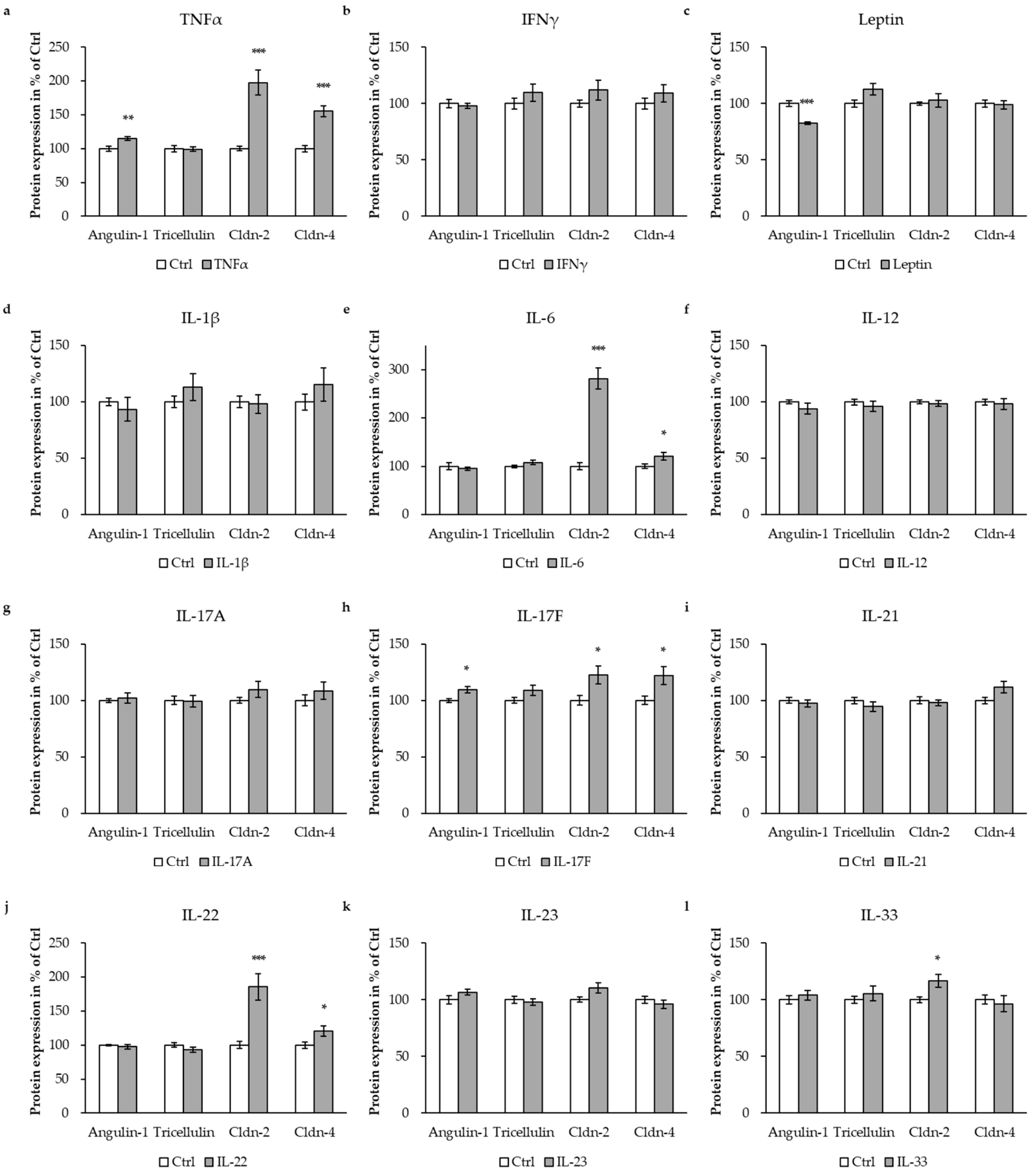

Figure S4 Cytokines effect on TJ protein expression in Caco-2 cells. After 48 h treatment with leptin, angulin-1 is downregulated to 82.38 ± 1.03 % (**c**, ***$p < 0.001$, n = 12). On the contrary, TNF-α and IL-17F slightly upregulated angulin-1 after 24 h (**a**, **$p < 0.01$, n = 12; **h**, *$p < 0.05$, n = 9).

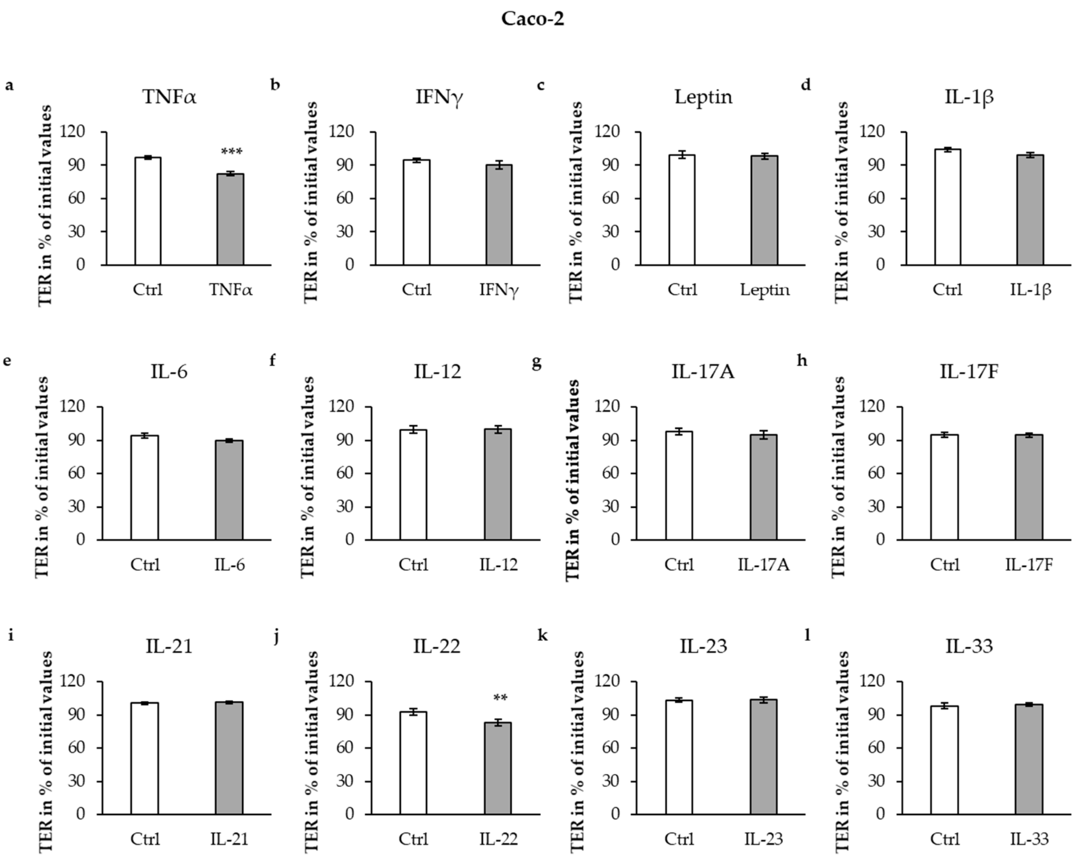

Figure S5 Cytokines effect on TER in Caco-2 cells. The TER of Caco-2 cells is decreased after 24 h of TNF-α treatment (**a**, ***$p < 0.001$, n = 9) as well as 48 h treated with IL-22 (**j**, **$p = 0.001$, n = 9).
